# Galvanizing Action on Primary Health Care: Analyzing Bottlenecks and Strategies to Strengthen Community Health Systems in West and Central Africa

**DOI:** 10.9745/GHSP-D-20-00377

**Published:** 2021-03-15

**Authors:** Aline Simen-Kapeu, Maria Eleanor Reserva, Rene Ehounou Ekpini

**Affiliations:** a United Nations Children's Fund, West and Central Regional Office, Dakar, Senegal.

## Abstract

**Introduction:**

The renewed commitment to primary health care (PHC) presents an opportunity to strengthen health systems in West and Central Africa (WCA). Though evidence-based cost-effective interventions that are predicted to prevent up to one-third of maternal, newborn, and child health complications and deaths with universal coverage have been identified, more than 50% of people living in rural areas or from poor families still do not have access to these interventions in resource-constrained settings.

**Methods:**

We conducted a multicountry systematic analysis of bottlenecks and proposed solutions to strengthen community health systems through a series of collaborative workshops in 22 countries in WCA. Countries were categorized by their under-5 mortality rate (U5MR) to assess specificities related to reported challenges. We also reviewed existing data on selected health system tracer interventions to analyze country profiles.

**Results:**

The bottlenecks identified as severe or very severe were related to health financing (19 countries, 86%), essential medical technology and products (16 countries, 73%), integrated health service delivery (14 countries, 64%), and community ownership and partnerships (self-reported by 14 countries, 64%). Only the integrated service delivery was self-reported as a severe challenge by countries with high U5MR. The issue of human resources for community health was one of the least reported challenges.

**Conclusion:**

In WCA, strengthening community health systems as part of PHC revitalization efforts should focus on increasing health financing and innovative investments, strengthening the logistics management system, and fostering community ownership and partnerships. Countries with high U5MR should also reinforce integrated service delivery approaches through innovation. Government actions galvanized by global and regional ongoing initiatives should be sustained to ensure that no one is left behind.

## Abstract (precis)

In West and Central Africa, “leaving no one behind” requires strengthening community health systems by increasing health financing, improving supply chain system, and fostering community ownership and partnerships in all settings. Countries with high child mortality rates should improve service delivery through better integration. Galvanizing context-specific country actions is fundamental to improve primary health care services and move toward universal health coverage.

## Teaser

**Key Findings:**

- Challenges with health financing, essential medical products and technology, and community ownership and partnerships emerged as the severe or very severe health system bottlenecks that hampered the strengthening of community health systems, irrespective of mortality context.
- Country-led progress is possible. Potential strategies to overcome bottlenecks include increasing domestic allocation and leveraging innovative funding mechanisms for primary health care (PHC), integrating supply chain systems, and strengthening policy implementation with communities and local governments.
- Countries with high child mortality rates should improve service delivery through better integration.

**Key Implications:**

- Countries must seize the opportunities to systematically strengthen community health systems in their efforts to achieve universal health coverage.
- We must galvanize efforts to mobilize resources for effective PHC, which is reliant on strong community health systems to expand access to services and live no one behind.
- Community-based integrated programming should be reinforced to strengthen resilience, disease surveillance, and rapid responses to health crises, including infectious diseases like COVID-19.

Résumé en français à la fin de l'article.

## INTRODUCTION

In 2018, the international community reaffirmed its commitment to primary health care (PHC) in the Declaration of Astana.^1^ In this declaration, PHC was articulated as a cornerstone toward achieving universal health coverage (UHC) and the United Nations (UN) Sustainable Development Goals (SDGs). Global health leaders agree that building stronger PHC delivery systems, with emphasis on community-based systems, is required to provide context-specific and locally-adapted responses to the needs of marginalized and underserved populations.^2^^–^^4^ A community health system is the set of local actors, relationships, and processes engaged in producing, advocating for, and supporting health in communities and households outside of, but existing in relationship to, formal health structures.^3^

Although it may take time to build enough health facilities providing quality services to ensure that marginalized and vulnerable populations, including those living in rural and remote areas, are within walking distance of health facilities, community health workers (CHWs) connected to well-trained PHC teams can extend the reach of high-quality care to people who need it the most, right where they are. Studies conducted in low- and middle-income countries have shown that focusing on provision of health services at the community level not only leads to more efficient and equitable use of health resources and better health outcomes but also is a consistent component of strong, effective health systems.^5^^,^^6^

Considerable progress has been made in improving health and well-being over the past 40 years, with dramatic reductions in maternal, neonatal, and child deaths.^7^^–^^15^ A systematic analysis found a significant impact of community case management with antibiotics: 32% (RR: 0.68, 95%CI: 0.53, 0.88) reduction in acute respiratory infections mortality and 20% (RR: 0.80, 95%CI: 0.77, 0.83) reduction in all-cause mortality among children aged 1–4 years.^16^ Although evidence-based, cost-effective interventions have been identified that are predicted to prevent up to one-third of maternal, newborn, and child health (MNCH) complications and deaths with universal coverage,^17^^–^^19^ more than 50% of people living in rural areas or from low-income families face challenges to access these simple interventions in resource-constrained settings.^20^^,^^21^

Although interventions have been identified that are predicted to prevent up to one-third of MNCH deaths, more than 50% of people living in rural areas or from low-income families don't have access to these interventions.

Many countries, especially in West and Central Africa (WCA), are lagging far behind the health-related SDG targets, calling for increased action.^22^ WCA is one of the poorest regions in the world and is affected by violent extremism, armed conflict, hazardous events including epidemics like Ebola and coronavirus disease (COVID-19), and climate-related disaster risks. These trigger humanitarian crises that weaken already overwhelmed health systems. As a result, scarce resources are diverted from health to security priorities.^22^^,^^23^ Slow progress is also due to weak government leadership, inadequate integration of basic services, and low access to care and treatment. There is little investment in community health, leading to limited large-scale implementation and major gaps in coverage of community-based interventions.^24^

However, the achievement of expected results varies between and within countries. There seems to be a difference between high mortality and low mortality countries in terms of health system functionality, capacity, and coverage of interventions.^25^^,^^26^ Knippenberg et al. noted that the strengths and weaknesses of a health system are crucial but are often not assessed in health program design, including community health.^26^ The big challenge remains how to put health systems strengthening into practice at the community level to achieve high, equitable, and effective coverage of care.

The big challenge remains how to put health systems strengthening into practice at the community level to achieve high, equitable, and effective coverage of care.

To ensure all populations have access to and use quality health services, systematic and context-specific identification of the health system barriers is needed to plan and implement community health programs. In this article, we aim to identify common community health system bottlenecks, review progress made by selected countries, and propose strategies to move forward. We also assess particularities by child mortality context through a multicountry analysis in WCA.

## METHODS

Between January and April 2019, we contacted all 24 countries in the United Nations Children's Fund (UNICEF) WCA region^27^ to conduct a systematic analysis of their community health system. We excluded Gabon and Sao Tome and Principe in the regional analyses as they reported not having a national community health policy or strategy (neither as a separate document nor clearly embedded in the national health strategic plan) or a clear framework describing the national community health program at the time of the survey. This was the only exclusion criteria. We then performed 2 analyses as followed.

### Analysis 1. Systematic Analysis of Bottlenecks to Strengthen Community Health Systems

#### Community Health System Bottleneck Analysis Tool

To assist countries in their analysis of health system bottlenecks, including identifying challenges that prevent the scale-up of community-based interventions and potential solutions, we developed the community health system bottleneck analysis tool, taking into consideration the World Health Organization (WHO)/UNICEF draft PHC operational framework (Supplement).^2^ The tool development involved: (1) adapting the health system framework used by Dickson et al. for the maternal-newborn bottleneck analysis tool,^25^^,^^26^ and (2) using the programmatic components described in the interagency Community Health Worker Assessment and Improvement Matrix (CHW-AIM)^28^ to assist data collection, compilation, analysis, and cross-country comparison. The bottleneck analysis tool, which is a questionnaire, was divided into 7 health system building blocks: (1) leadership and governance (including policies and coordination), (2) health financing, (3) human resources, (4) essential medical technologies and products, (5) health service delivery (and quality of care), (6) health information systems, and (7) community ownership and partnership.^29^ The seventh building block, community ownership and partnership, was included on the basis of the recommendations of the Ouagadougou Declaration on PHC.^30^ The tool was tested in 2 countries and slightly revised.

To better analyze supply chain challenges, we considered the recommendations of the United Nations Commission on Life-Saving Commodities to improve access to essential commodities.^31^^,^^32^ We considered the following areas: (1) availability of policy or strategy, (2) finances, (3) efficiency regulation, (4) product quality and patient safety, and (5) procurement and availability of inputs. Second, we selected the 3 recommended essential medicines that are required to treat the major causes of child deaths: amoxicillin, oral rehydration salts, and zinc, as well as artemisinin-based combination therapy.

#### Participants and Process for Country Consultations

The community health bottleneck analysis tool was used in a series of national workshops held between January 15 and April 30, 2019, in the 22 selected countries. The number of workshop participants varied by country and included members of national technical working groups that consisted of program managers from the ministries of health, UN agencies, nongovernmental organizations, bilateral agencies, and other stakeholders at national levels. Members of the working groups were experts from diverse fields nominated by their governments to provide advice on community health issues on a regular basis. The workshop brought the working group members together to assess bottlenecks and propose strategies to strengthen community health systems. More than 200 individuals participated in these ministry of health-led workshops. Participants were oriented on the use of the tool during the first day of the workshop.

Participants examined each of the 7 health system building blocks—based on data and experience—to identify the key challenges. The groups then came to a consensus on whether the bottlenecks to the health system area should be graded as good (not a bottleneck), needs minimal improvements (minor bottleneck), needs important improvements (severe bottleneck), or inadequate (very severe bottleneck). Finally, participants proposed potential strategies to address priority challenges identified. The ministry of health program managers and working group members were responsible for collating all responses and submitting the final data; they also served as points of contact for clarification of any issues. In November 2019, the first high-level regional forum on community-based PHC organized in Benin was an opportunity to further discuss and validate the results with the 13 country teams who participated in the meeting.

Workshop participants used the community health bottleneck analysis tool to examine the 7 health system building blocks and identify challenges.

#### Data Analysis and Grading of Bottlenecks

We received complete national-level data from 22 countries. We reviewed all the bottlenecks for each health system building block and all solutions presented by country participants (Tables 1 and 2). Issues reported by at least 3 countries were further reexamined against recent country surveys^14^ to assess their persistence. From all bottlenecks, we extracted those that workshop participants categorized as severe or very severe to establish whether there were health-system areas that could be prioritized to move forward. For more context-specific subanalysis, we categorized the bottleneck analysis data from the 22 countries into 2 under-5 mortality rate (U5MR) categories: U5MR of more than 75 deaths per 1,000 live births and U5MR between 25 and 75 deaths per 1,000 live births. A health system block is defined as a priority if at least 50% of the reporting country teams graded the health system area as severe or very severe. We also reviewed all strategies proposed by country teams to address identified challenges and proposed a grouping by country typology whenever possible.

**TABLE 1.** Country Self-Grading of Health System Domains as Severe/Very Severe^a^ Bottlenecks to Strengthen Community Health Systems (N=22 Countries^b^)

	Legislation, Policies, Governance, Coordination	Health Financing	Essential Medical Technology and Products	Human Resources	Service Delivery and Integration; Quality of Services	Information System/ Monitoring and Evaluation	Community Ownership andPartnership
Benin	*				*		*
Burkina Faso		*	*		*	*	*
Cameroon	*	*	*	*		*	*
Cabo Verde			*				
Central African Republic	*	*	*	*	*	*	*
Chad			*		*		
Congo	*	*	*	*			*
Cote d'Ivoire	*	*	*		*		*
Democratic Republic of the Congo		*	*		*		*
Equatorial Guinea	*	*			*	*	
Gambia	*	*	*	*	*		
Ghana							
Guinea		*	*	*	*	*	*
Guinea-Bissau		*			*		
Liberia		*	*		*		
Mali		*					
Mauritania	*	*	*		*	*	*
Niger	*	*	*			*	*
Nigeria		*	*	*	*	*	
Senegal		*	*	*			*
Sierra Leone	*	*	*		*		*
Togo		*	*				*
**Total number of countries**	10/22	19/22	16/22	7/22	14/22	9/22	14/22

a * indicates severe/very severe.

b Excluding Gabon and Sao Tome and Principe as described in the method section.

**TABLE 2.** Country Self-Grading of Health System Domains as Severe/Very Severe^a^ Bottlenecks to Strengthen Community Health Systems by Under-5 Mortality Rate Category (N=21 Countries^b^)

Countries/Domains	Legislation, Policies, Governance, Coordination	Health Financing	Essential Medical Technology and Products	Human Resources	Service Delivery and Integration; Quality of Services	Information System/ Monitoring and Evaluation	Community Ownership and Partnership
**Countries with under-5 mortality rate = 25 to 75 deaths/1,000 live births (6 countries)**							
Congo	*	*	*	*			*
Gambia	*	*	*	*	*		
Ghana							
Liberia		*	*		*		*
Senegal		*	*	*			*
Togo		*	*				*
**Total number of countries**	2/6	**5/6**	**5/6**	3/6	2/6	0/6	**4/6**
**Countries with under-5 mortality rate > 75 deaths/1,000 live births (15 countries)**							
Benin	*				*		*
Burkina Faso		*	*		*	*	*
Cameroon	*	*	*	*		*	*
Central African Republic	*	*	*	*	*	*	*
Chad			*		*		
Cote d'Ivoire	*	*	*		*		*
Democratic Republic of the Congo		*	*		*	*	*
Equatorial Guinea	*	*			*	*	
Guinea		*	*	*	*	*	*
Guinea-Bissau		*			*		
Mali		*					
Mauritania	*	*	*		*	*	*
Niger	*	*	*			*	*
Nigeria		*	*	*	*	*	
Sierra Leone	*	*	*		*		*
**Total number of countries**	8/15	**13/15**	**11 /15**	4/15	**12/15**	9/15	**10/15**

a * indicates severe/very severe.

b Excluding Gabon and Sao Tome and Principe as described in the method section. Cabo Verde has less than 25 deaths/100 live births.

### Analysis 2. Quantitative Analysis of Country Profiles With Selected Tracer Indicators

To complement the bottleneck analysis in the 22 countries in WCA, we selected tracer indicators from the health system building blocks and indicated the coverage of key indicators for children to analyze countries' progress toward reducing child mortality from a multisectoral lens. We categorized countries according to the latest U5MR, relative to one of the targets for the third Sustainable Development Goal (SDG3), which is to end preventable deaths of children under 5 years old by 2030, with all countries aiming to reduce under-5 mortality to at least 25 per 1,000 live births (LBs). Based on this target we proposed 3 groups of countries: Group 1 countries had U5MR of 25 deaths per 1,000 LB or less, Group 2 countries had U5MR between 25 and 75 deaths per 1,000 LB, and Group 3 countries had more than 75 deaths per 1,000 LB.

We further analyzed health system tracer indicators from existing global data to assess the profile of countries studied, taking into consideration the health system building blocks. Selected indicators can be found in Table 3. Data were most available on health facility service delivery, while data on essential medical products and technology were limited, with only 8–9 countries reporting on the chosen indicators. Variations were also found within health financing—data on national health financing was complete for all 22 countries but became less available for PHC-level financing data. The list of indicators included 5 indicators on health financing, 4 indicators on essential medical products and technology, 2 indicators on health workforce (focused on community health), and 4 indicators on health facility service delivery. To complement the analysis, tracer indicators for child health interventions and child health-related multisectoral interventions that address overlapping children's deprivations were also added.

**TABLE 3.** Grouping of 22 West and Central African Countries According to Under-5 Mortality Rates Showing Selected Health System Tracer Indicators (Financing, Supply, Health Workforce, and Service Delivery) and Child Indicators

	Group 1, U5MR <25 Deaths per 1,000 Live Births (N=1)^a^	Group 2, U5MR 25 to 75 Deaths per 1,000 Live Births (N=6)^b^	Group 3, U5MR >75 Deaths per 1,000 Live Births (N=15)^c^	Countries With Data,No.
**Mortality/demographics^d^**				
Under-5 mortality rate (Median, deaths per 1,000 live births)	19.5	54.2	88.1	22
Annual rate of reduction (2000–2018)	3.3%	4.2%	3.3%	22
**Health financing^e^**				
Median primary health care expenditure as % current health expenditure (2016–2017)	63.3%	67.7%	73.5%	14
Median primary health care expenditure per capita (2016–2017)	US$94.5	US$34.9	US$24.7	13
Median domestic general government health expenditure per capita, PPP (2017)	US$215.1	US$34.5	US$25.7	22
Median government expenditure on health, percentage of gross domestic product (2018)	3.1%	1.5%	1.0%	22
Median out-of-pocket expenditure, percentage of current health expenditure (2017)	28.9%	48.5%	43.5%	22
**Supply^f^**				
Median essential drug availability	-	37.1%	29.0%	9
Median vaccine availability	-	83.9%	76.5%	8
Median basic equipment availability	-	87.0%	82.0%	9
Median facilities with clean water, electricity, & sanitation (% of health facilities)	-	54.2%	69.2%	8
**Health workforce**				
Median community and traditional health worker density (per 1,000 population)	-	0.1	0.1	10
Median health center density (per 100,000 population)	3.8	5.1	4.4	15
**Health facility service delivery^g^**				
At least 1 antenatal visit, median	97.6%	92.0%	83.2%	22
At least 4 antenatal visits, median	72.3%	77.9%	51.3%	22
Skilled attendance at birth, median	92.3%	64.8%	67.3%	22
Institutional delivery, median	75.6%	72.8%	66.8%	22
**Child health tracer indicators^h^**				
Diphtheria-tetanus-pertussis immunization coverage, median	98.0%	86.0%	79.0%	22
Children with diarrhea treated with oral rehydration salts, median	-	36.0%	34.3%	21
Children with malaria with first-line treatment (artemisinin-based combination therapy) for under-5, median	-	57.8%	17.5%	21
Children with symptoms of acute respiratory infection who received antibiotics, median	-	40.2%	27.8%	21
**Other child health-related tracer indicators (multisectoral)^h^**				
Children (0–14) living with HIV, median no.	-	6,100	10,000	21
Children (0–14) living with HIV receiving antiretroviral therapy, median	-	27.5%	23.4%	21
Children under 5 years who are stunted (moderate and severe), median	21.0%	23.0%	32.0%	22
Children under 5 years who are wasted (severe), median	-	1.5%	2.0%	21
Children under 5 years who have their births registered, median	91.4%	74.7%	65.6%	22
Households with at least basic drinking water services, median	87.1%	75.6%	63.3%	21
Households with at least basic sanitation services, median	73.9%	19.3%	21.6%	21
Households with at least basic hygiene facilities, median	-	17.3%	14.7%	18

Abbreviation: U5MR, under-5 mortality rate.

a Cabo Verde.

b Senegal, Ghana, Congo, Gambia, Togo, Liberia.

c Mauritania, Cameroon, Burkina Faso, Cote d'Ivoire, Guinea-Bissau, Niger, Equatorial Guinea, Democratic Republic of the Congo, Benin, Mali, Guinea, Sierra-Leone, Central African Republic, Chad, Nigeria.

d Mortality/Demographics Source: 2019 United Nations Inter-agency Group for Child Mortality Estimation (United Nations Children's Fund, World Health Organization, United Nations Population Division and the World Bank Group).

e Health Financing Sources: Databank from The World Bank, Global Health Expenditure Database from World Health Organization, and Primary Health Care Performance Initiative.

f Supply Source: Based on country's latest results from the Service Availability and Readiness Assessment, if available.

g Health Workforce Source: Primary Health Care Performance Initiative.

h Health Service Delivery, Child Health, Multi-sectoral Child Needs: Based on latest Demographic and Health Survey or MICS Country Survey Results and 2019 State of the World's Children Report.

## RESULTS

### Self-grading of Bottlenecks by Country Teams

A total of 22 country teams identified and self-graded the bottlenecks to strengthen community health systems in their respective country contexts. Tables 1 and 2 summarize the overall grading across all the countries, as well as grouped by U5MR.

Table 1 shows that for all 22 countries, the bottlenecks most frequently identified as severe or very severe (affecting at least 50% of the countries) were health financing (self-reported by 19 countries or 86%), essential medical technology and products (self-reported by 16 countries or 73%), integrated health service delivery (self-reported by 14 countries or 64%), and community ownership and partnerships (self-reported by 14 countries or 64%).

We conducted subanalyses of these bottlenecks by further categorizing countries according to their mortality context. As displayed in Table 3, only 1 country met the criteria for Group 1, 6 countries met the criteria for Group 2 (median U5MR of 54.2 deaths), and 15 countries met the criteria for Group 3 (median U5MR of 88.1 deaths). Table 2 shows that for the country teams with U5MR over 75 deaths per 1,000 live births (15 countries), health financing (13 countries) was the dominant challenge, followed by essential medical technology and products (11 countries), and community ownership and partnerships (10 countries). The major difference between both settings was that integrated service delivery at the community level was not a major concern for country teams in settings with U5MR between 25 and 75 deaths per 1000 deaths (2 of 6 countries) compared to country teams in settings with U5MR above 75 deaths per 1000 deaths (11 of 15 countries).

The results from the grading patterns showed that overall, and irrespective of mortality context, health financing, essential medical products and technology, and community ownership and partnerships emerged as the health system building blocks that were consistently rated as having severe or very severe bottlenecks.

### In-depth Country Review of Bottlenecks

Further analysis of the thematic areas (Supplement, Table 1) self-reported by country teams (at least 50% of countries) revealed that commonly, health financing challenges were due to the lack of a budget line for community health or PHC (13 countries), inefficient financial flows (13 countries), and mostly, the absence of a clearly defined resource mobilization pathway outlining funding gaps and potential funding sources that could be tracked and collected (20 countries).

The second most challenging health system block was essential medical technology and products, which was self-reported by 16 of 22 countries, had several underlying issues. Countries highlighted lack of training of health facility staffs (17 countries), frequent stock-out of selected drugs (15 countries), nonfunctional monitoring tracking system for drugs on a real-time basis (15 countries), and inadequate or lack of quantification of community needs as part of the national annual quantification exercise (14 countries).

Community ownership and partnerships was self-reported by country teams as a weak area exacerbated by the lack of a national community engagement strategy (13 countries), inadequate linkages between CHWs and community members (13 countries), and the absence of functional mechanisms for social accountability and citizen engagement (14 countries). In countries with U5MR over 75 deaths per 1,000 LB, integrated service delivery was a challenging building block mainly due to poor integration between MNCH and TB/HIV, birth registration, promotional and preventive adolescent sexual and reproductive health services, or early childhood and education. Less than 40% of country teams reported issues integrating MNCH and nutrition or water, sanitation, and hygiene interventions.

### Progress in Strengthening Community Health Systems

Despite the observed challenges, countries made progress during the past 5 years (Supplement, Table 2). We highlight selected country-led efforts toward strengthening their community health systems in the context of PHC with the support of stakeholders working in the field of health system strengthening. This nonexhaustive list of selected achievements includes: Primary Health Care Development Agency Act (Nigeria), Primary Health Care reform (Mali), Integration of the community health module into the district health information system or DHIS2 (Senegal, Liberia), National Health Insurance financing scheme (Ghana), and financial contribution of local collectivities/government through their annual investment plan (Guinea), to name a few (Supplement, Table 2).

Table 4 presents the strategies proposed by country teams to address the bottlenecks hampering community health system strengthening. Whenever possible, we have proposed specific actions for countries in Groups 1 and 2 with a stronger health system than those in Group 3. The working group members conceptualized these solutions by health system areas and according to individual country contexts, yet several common solutions emerged. For example, the development of an investment case for community health or for PHC (which includes the community component) could better inform resource mobilization efforts, and fostering implementation and expansion of pro-poor legislation and strategies could reduce financial barriers limiting access to services. Countries are also encouraged to establish functional mechanisms for social accountability (community scorecards and observatories) and citizen engagement with communities to enhance quality care. We further propose taking a closer look at the key strategic priorities to be jointly (with communities) addressed in each of the 7 countries that are part of the Community Health Roadmap initiative^9^ in WCA (Table 5).

**TABLE 4.** Examples of Country Achievements and Proposed Strategies to Strengthen Community Health Systems

	Highlights of Recent Achievements	Key Strategies to Enhance Current Efforts
**Group 1 and 2: Countries with U5MR < to 75 deaths / 1,000 live births**		
**Senegal**	High-level commitment and government leadership to scale up PHC: national health financing strategy developed; National UHC program defined; PHC review completed (2018–2019) National community health program 2019–2023 National health investment case developed; including community health component Various CHW cadres in place, mostly funded by external resources Engagement of local authorities for the management of health posts (case de santé) through local health development committee Ongoing resource mobilization efforts conducted, with a great focus on external resources CHW package of services defined; scale up of integrated services in most regions, including health and nutrition at the community level CHIS developed and integrated into DHIS2	Increase domestic resources mobilization for community-based PHC by leveraging investments from local governments or other ministers (education, youth); Leverage innovative funding mechanisms: education and economic growth programs; co-financing/matching funds, trust funds with private sector Implement performance-based financing based on lessons learned from the pilot program and local contexts Work toward professionalizing CHWs to strengthen health systems Strengthen community-based logistic management system (forecasting, procurement, quality control) as well as last-mile distribution of essential drugs Scale up innovative technologies for better health management system, including information and logistics management systems Enhance social accountability mechanisms (community scorecards, observatories) and citizen engagement for better planning and monitoring with local communities Invest in the building and equipping health posts (case de santé) close to the communities in the deprived regions of the country
**Liberia**	High-level presidential engagement to scale to community health post Ebola virus disease-crisis (2016) Community health investment case completed; National community health policy developed Financing gap analysis and resource mobilization, with a main focus on external funding – international aid Roll-out of the national community health assistant program: training modules and tools developed; community health assistants and supervisors recruited and trained in selected regions CHIS is integrated into DHIS2; Ongoing digitalization of the system to strengthen CHW performance	Strengthen the functionality of national coordination and governance mechanisms for PHC including community health Review of national community health policy as per WHO recent guidelines Build a pathway toward financial sustainability: develop clear gap analyses and financing pathways for the CHA program by leveraging domestic/ international resources, new funding sources including matching grants, co-investment with local governments as well as the private sector Work with local governments and other line ministries (decentralization, social welfare, youth, education) to ensure long-term availability of community health assistant (remote and rural areas) Fully integrate community-based LMIS into national LMIS and improve last-mile distribution of drugs Generate evidence on innovative approaches and best financing models for the national CHW program Develop referral systems with local governments and communities Enhance community governance and oversight mechanisms for the community health assistant program; establish community-based monitoring and social accountability systems
**Group 3: Countries with U5MR Over 75 deaths/1,000 live births**		
**Guinea**	National health investment case developed; National community health policy developed (CHA profile harmonized) National intersectoral coordination mechanism established, under the leadership of the Ministry of Territorial Administration and Decentralization to support implementation of the national community health policy Resource mobilization through the National Financing Agency for local collectivities; financial contribution of local collectivities through their annual investment plan Implementation of the national CHA program in selected districts (mainly funded by donors): development of tools, training of CHAs Engagement of community leaders and networks in decision making and monitoring enhanced through local government structures and local committees Development of the CHIS (tools and modules)	Raise awareness on specific community health policy issues (parliament, champions) Implement and expand pro-poor legislation and strategies (e.g., vouchers, community-based health insurance schemes) Develop clear gap analyses and financing pathways for costed CHW programs by leveraging domestic/international resources, new funding sources including matching grants, co-investment with local governments, disease surveillance and preparedness Leverage/strengthen innovative partnerships, especially with the private sector to scale up community health services Work toward integrating CHWs as part of the PHC multidisciplinary team and expand quality improvement mechanisms to increase CHW performance Develop and implement innovative and digital approaches to strengthen data collection and use at the community level Fully integrate community needs into the logistics management systems and ensure last-mile distribution of products Strengthen referral system between communities and health posts/facilities
**Sierra Leone**	High-level commitment at the presidential level for the UHC program; Review of PHC services completed CHW governance structure in place; Community health investment case developed (2017) Community health program review completed (2019); ongoing review and development of PHC model of care (2019-2020) Free Health Care initiative with essential commodities (includes amoxicillin, oral rehydration solution-zinc) Harmonization of CHW service delivery package, including the expansion of services to be provided by CHWs Deployment of a large number of more than 15,000 various CHW cadres under the leadership of the government, with the support of partners Review of the CHIS modules and integration into the DHIS2	National health financing strategies should consider CHW systems within a broader framework of financing for UHC Mobilize domestic resource to support PHC services, including community health; Leverage/strengthen innovative partnerships, especially with the private sector to scale up community health services Review and harmonization of the CHW profile/cadre according to recent WHO guidelines; Mapping and redeployment of CHW as per recent needs assessment and equity analyses Work toward integrating CHWs as part of the PHC multidisciplinary team and expand quality improvement mechanisms Better integrated community needs into national logistics management information system and strengthen local system to ensure last mile distribution of drugs Develop digital approaches to strengthen data collection, analyses and use at the community level; roll out standard operating procedures for data quality Establish social accountability (scorecards, observatories) and citizen engagement mechanisms with communities

Abbreviations: CHA, community health agent; CHIS, community health information system; CHW, community health worker; DHIS, district health information system; PHC, primary health care; U5MR, under-5 mortality rate; UHC, universal health coverage; WHO, World Health Organization.

**TABLE 5.** Community Health Roadmap Initiative: Selected 2020 Country Priorities to Move Forward

The Community Health Roadmap is an innovative collaboration between multilateral and bilateral donors; private funders; and global health leaders, including USAID, the World Bank, WHO, Bill & Melinda Gates Foundation, The Rockefeller Foundation, UNICEF, and Office of the WHO Ambassador for Global Strategy, to better align existing resources and to attract new resources to community health and support countries in achieving their goals for PHC, UHC, and SDG3. The Roadmap aims to elevate national priorities and create a common agenda for investments in community health to strengthen primary health care. In West and Central Africa, 7 of 15 countries have been selected for initial inclusion in the Roadmap. The selected 2020 key priorities to move forward with community health systems strengthening efforts are listed below.	
Burkina Faso	Develop a clearly defined pathway to mobilize domestic resources for community health (financial gaps, potential sources of funding, and actions) Establish sustainable mechanism for the contextualization and remuneration of CHWs with the support of local governments and community leaders Expand community health posts as part of the PHC/UHC initiative
Central African Republic	Develop a community health investment case and return on investment analysis Review the community health policy as per 2018 WHO guidelines, taking into consideration the humanitarian-nexus development context Establish functional community health information and supply chain systems that will sustain the community health program (integrated into the national PHC system)
Côte d'Ivoire	Finalize the National Community Health Policy Operationalize the National Strategic Plan for Community Health Develop a clearly defined pathway to mobilize domestic resources for sustainable financing for CHW remuneration, in close collaboration with local municipalities and private sector Develop community health standards and procedures to harmonize implementation
Demographic Republic of Congo	Provide high-level advocacy to close financial gaps identified in the national community health investment case and resource mobilization plan Improve the coverage and functionality of community care sites (e.g., for iCCM) to cover at least 50% of needs and adding at least 3,484 community care sites across the country by 2022 Strengthen managerial and resilience capacities of community outreach units to contribute to national health security priorities, taking into consideration the gender dimension Integrate community health information system into the DHIS2
Mali	Revise the community health strategic plan, in line with the 2018 WHO guidelines, taking into consideration the humanitarian-nexus development context Support the free care-costing analysis to advocate for resource mobilization and improve equity access of the vulnerable population to quality care, in close collaboration with local municipalities and private sector Expand the community platform model to strengthen community partnerships and leadership in the implementation of the CHW program, taking into consideration the gender dimension Establish a functional community health information system integrated into the DHIS2, and capitalize on use of digital tools to improve program performance
Niger	Develop an investment case and financing gap analysis to guide resource mobilization efforts Conduct a detailed partner mapping to support financing and operationalization of the strategic plan Support ongoing efforts to strengthen procurement systems to include the community dimension and eliminate parallel structures Integrate community health information system into the DHIS2

Abbreviations: CHW, community health worker; DHIS, district health information system; iCCM, integrated community case management of childhood diseases; PHC, primary health care; UHC, universal health care; UNICEF, United Nations Children's Fund; USAID, U.S. Agency for International Development; WHO, World Health Organization.

## DISCUSSION

The renewed commitment to PHC^1^ presents an opportunity to optimize the functionality of community health systems and accelerate progress toward universal health coverage and SDGs. Functional PHC systems provide adequate care with the communities and through the communities, ensure preparedness against future epidemics, fight against the major causes of deaths, and build capacity to handle the growing burden of non-communicable diseases.

This study is the first multicountry analysis of bottlenecks and strategies to strengthen community health systems in WCA. There is a growing body of literature synthesizing current evidence and developing conceptual understandings on the design of national CHW programs and the processes of scaling up and integration into national health systems.^3^ Despite recent progress, substantial efforts are required by all to accelerate progress.^32^ This study emphasizes the great need to focus on increasing health financing, strengthening the supply chain system, and fostering community ownership and partnerships to strengthen community health systems in all settings. Countries with high U5MR should also reinforce integrated service delivery approaches through innovation.

This study emphasizes the need to improve health financing, strengthen the supply chain system, and foster community ownership and partnerships to strengthen community health systems in all settings.

### Increase Health Financing

Our study shows that health financing was a very severe hurdle in all country contexts. Financing PHC or CHW programs have continued to be a major obstacle to improving health outcomes in Africa,^33^ particularly considering that the WCA region bears the bulk of the global morbidity and mortality burden for mothers, newborns, children, and those infected by HIV.^22^ One explanation could be that government health prioritization did not seem to be associated with national income or level of government revenues in Africa.^34^ Despite increases in fiscal capacity in some countries, spending on health as a proportion of total public expenditure had been de-prioritized as governments strived to meet other obligations; and this tended to be associated with country-level fragility or poor governance.^34^ The Health Systems Strengthening Accelerator^11^ is partnering with U.S. Agency for International Development missions, country leaders, and partners in Cote d'Ivoire, Guinea, and Togo to develop near-term adjustments to public financing to lay the foundation for longer-term transition and ensure adequate and efficient use of health sector resources.

Another explanation could be that, in most African countries, public monies flowed disproportionally to high-end care at secondary and tertiary levels, referral hospitals, and capital facilities.^33^^,^^34^ Because of prohibitive costs also, governments balked at optimizing CHW programs through their proper integration in health systems. Taking into account the challenges of raising sufficient domestic resources for health, distributing the burden of health expenditure in an equitable manner, and addressing the need for efficient use of the scarce resources, close collaboration among the ministries of finance, territorial or internal affairs, and health is vital.^32^ External funds will still remain critical in many contexts, but efforts should focus on improving predictability of funding flows and harmonizing funds allocation with national priorities and mechanisms to ensure their effective use. The Financing Alliance for Health^13^^,^^35^ helps governments design and fund ambitious, affordable, and at-scale community health programs including finding innovative financing pathways and investment opportunities that utilize the private sector. Technical assistance has also been provided to countries, including in WCA, to develop community health strategies, comprehensive community health packages, community health investment cases, community health model improvement interventions, and financing policy briefs to advocate for resource mobilization.

### Strengthen the Supply Chain

Most country teams self-reported that the supply chain was a severe health system bottleneck due to reasons such as lack of funding including for operating costs, ineffective procurement cycles and delays, inadequate commodity security strategies, and inadequate quantification for CHWs. Country-specific implementation barriers could have been overlooked due to information gaps and lack of data sources in this area. However, the literature showed that access to medicines or health commodities remained one of the most serious global public health problems and resulted in critical gaps in the delivery of PHC services.^36^ Challenges reported by country teams were also most likely due to heterogeneity in the governance structure of central medical stores, existing parallel systems that are not government-led, non-inclusion of the community module into the national supply chain plan causing inadequate budgeting, and poor health worker performance and accountability. Similar findings were observed in various countries in Africa.^37^^,^^38^ In addition, Pronyk et al.^31^ showed similar supply chain system bottlenecks in relation to the accessibility and availability of the 13 reproductive care and MNCH lifesaving commodities.

For all settings, key strategies to address challenges could include: effective integration of the community-based supply chain system into national supply chain policies, systems, training on data collection and analysis leading to improved forecasting and reduced stock-outs, reducing tiers in the operational system, streamlining information flows, using of mobile technology across tiers and/or facilities where possible, and developing efficient quality assurance processes.

Additional challenges were absent or irregular supplies of key commodities in public health facilities (including CHWs) most likely due to weak systems for restocking, inadequate quantification, or lack of funds. One strategy could be the use of information and communication technology (ICT) or computerized systems that analyze local data use to drive the supply of commodities according to need, which can be set to both forecast seasonal needs and to generate alerts when commodities fall below a specified threshold.^37^^,^^39^ A hallmark of functioning health systems is the availability of essential medicines in adequate amounts, appropriate dosage forms, assured quality, and at a price that is affordable for local communities.^40^ Because most health care delivery occurs at the lowest level of care, efforts should focus on ensuring a more responsive integrated supply chain management system to substantially improve the effectiveness of health workers.

Efforts to strengthen the supply chain should focus on ensuring a more responsive integrated supply chain management system to improve the health workers' effectiveness.

### Foster Community Engagement and Partnerships

Community engagement was a severe challenge for most country teams in all contexts. Enhancing community participation—a fundamental principle of PHC—has proved problematic, and how it is operationalized and sustained in practice is not always well understood.^41^^,^^42^ Our study, as well as previous studies,^14^^,^^41^^,^^43^ identified underlying issues such as the lack of national community engagement strategy, limited social capital and community capacities, and absence of national and local established mechanisms to enhance local stakeholders' accountability. Many international health policies recognize the WHO's vision^30^ that communities should be involved in shaping PHC services. The literature revealed a small but substantial body of evidence that community engagement is associated with improved health outcomes.^44^ For example, in Burkina Faso, Mali, and Côte d'Ivoire, as a matter of policy,^14^ respective governments aim at increasing access to health services through CHWs, civil society organizations, women's groups, nongovernmental organizations through community partnership, and full participation. Additional research on the impact of community-based participatory processes remained to be done at a large scale in WCA. In other settings, findings showed that partnering with communities could lead to designing new service models that fit within existing budgets and address local aspirations and health care priorities.^45^ Country teams proposed that policy makers should develop community engagement plans, strengthen policy implementation with communities, and support funding for participatory mechanisms in PHC.

### Integrate Service Delivery

The highest burden of mortality and morbidity is often seen where health system gaps are the greatest.^25^^,^^26^^,^^46^ Since mortality data can be regarded as a tracer for the health system, countries were split into categories by U5MR. Countries with higher U5MR (Group 3) reported additional challenges related to integrated service delivery most likely due to poor coordination across programs, inadequate professional skills, or lack of cross-sectoral funding mechanisms. Our analyses also showed that these countries had lower government expenditures on health and lower intervention coverage than those with lower mortality rates.

Few country teams reported challenges in integrating MNCH and integrated community case management of childhood diseases.^47^ Poor MNCH/HIV/TB service integration was reported as a severe bottleneck by 14 countries. The majority of the missing cases with TB and HIV will only be found through decentralized, integrated, and family-centered service delivery at the PHC level. Screening and awareness of TB and HIV need to be an integral part of community-based child health and nutrition programming in high-burden settings, with shared mandates and accountabilities across health programs.^48^^–^^52^ Integrated case management of multiple diseases by appropriately trained CHWs has been demonstrated to be feasible, promote care seeking, improve rational antibiotic use, and reduce all-cause mortality among children under 5 years old.^24^^,^^47^^,^^52^^–^^54^ A Cochrane review found that integrated management of childhood illnesses was associated with a 15% reduction in child mortality when activities were implemented at both health facilities and communities.^15^

Community-based integrated programming should be reinforced to strengthen disease surveillance, rapid responses to health crises that may emerge in different areas, including infectious diseases (like coronavirus disease [COVID-19]), first aid, and mental health as we learned from the Ebola outbreak.^55^ Whereas similar investigations have examined health system challenges to the scale-up of newborn care,^25^ pneumonia and diarrhea,^56^^,^^57^ and malaria treatment,^58^ this analysis provides new insight into which system bottlenecks are severe and common for community-based health care in WCA.

Community-based integrated programming should be reinforced to strengthen resilience, disease surveillance, and rapid responses to health crises, including infectious diseases like COVID-19.

### Strengthen Leadership and Governance, Health Information System, and Human Resources

#### Leadership and Governance

Although this health system block has not been self-graded as a major bottleneck by country teams, its importance cannot be overstated. Governments and communities need to plan and act to institutionalize CHWs as per the 2018 WHO policy^59^ since relying on donor funding is not sustainable. Communities are crucial drivers for health system efforts to scale up and improve care and need to be involved in all mortality contexts. Country teams also highlighted the need for operational changes to support service integration, including tailoring actions and resources to reach the most disadvantaged areas and social groups and building capacity in PHC to deliver proactive promotion and preventive care at the community level.

In terms of partnerships, there is also a need for strong intersectoral linkages beyond health alone as well as multilevel partnerships, which are crucial to driving and maintaining effective systems. More countries in WCA can also benefit from global and regional ongoing initiatives to mobilize investments and expertise to strengthen community health systems including the Global Financing Facility,^7^ the 2017 Institutionalizing Community Health Conference,^8^ the Community Health Roadmap,^9^ the 2019 Primary Health Care Conference, the Primary Health Care Performance Improvement partnerships,^10^ the Health System Strengthening Accelerator,^11^ the Health Data Collaborative, the Collectivity,^12^ the integrated Community Case Management Financing Task Team, and the Financing Alliance for Health.^13^ All these initiatives aim to support governments in their efforts to strengthen the health system through health policy reforms and initiatives to increase government expenditures on health and expand basic services to all, including the most vulnerable.

#### Health Information System

Although this building block was not self-graded a major bottleneck, at least 50% of countries reported challenges related to lack of community data, poor quality of data reported, and the capacity of communities to analyze and use the data for decision making. This is most likely due to weak supervision of CHWs by health facility staff and insufficient number of CHWs trained on information system management. Several countries, including Liberia, Sierra Leone, Ghana, and Senegal improved their information system by integrating the community health module into the district health information system (DHIS2). In addition, Ghana moved forward with the roll-out of eHealth.^60^^,^^61^ Additional efforts should be done to develop and train PHC staff, including CHWs, on standard operating procedures for data management and use. Although eHealth has the ability to positively influence the quality of health care and improve health services, there are a number of challenges to its adoption.^60^ Constraints to the adoption of eHealth in Africa include the low ICT budgets, poor infrastructure in support of health services, erratic electricity supply, and inadequate human resource capacity.^60^^,^^62^ The private sector's involvement in spearheading an eHealth revolution within the subregion could be an immense benefit to alleviating the burden on governments and their inadequacy.^60^

#### Human Resources

Challenges related to human resources at the community level were not perceived to be severe or very severe by most country teams, most likely due to ongoing efforts. A regional study conducted by WHO in 2019 showed that 70% of 47 African countries had a human resources strategy or plan showing their commitment to address workforce issues.^59^^,^^63^ Although these plans particularly emphasized equitable distribution of health workers to rural and hard-to-reach areas and the use of incentives to recruit and retain them to those areas, only 8% of plans mentioned formally integrating CHWs into the health system to meet the CHW shortfall or to train and integrate them to assure equitable distribution of CHWs throughout the country. This is in line with our study in which most country stakeholders reported the lack of retention and motivation mechanisms (12 countries) or any forms of contracts (17 countries) for CHWs. A recent review found strong support for ensuring community embeddedness, as this was associated with CHW retention, motivation, performance, accountability, and support and ultimately affects the acceptability and uptake of CHWs' health-related work.^64^ In the WCA region, CHWs are mostly male; not only gender-responsiveness of policies is still inadequate, but also questions about these unbalanced ratios are insufficiently raised.^14^

### Strengths and Limitations

We categorized bottlenecks by health systems building blocks to allow the identification of issues and implementation of solutions. However, we recognize that barriers to care are inter-related and their solutions cut across several building blocks.^25^ For instance, low demand for care could be due to current nonavailability of services (health workforce),^33^ affordability (health financing),^65^ or lack of community awareness (community ownership and partnership).^66^ The focus on child health interventions for some health system areas may warrant some further narrowing of the presentation of findings, as issues around community-based child health interventions are not necessarily transferable to other community health areas. Country bottleneck identification depended upon the generation of categorical variables from nonstandard and qualitative data collected by different enumerators. Despite the use of expertly defined performance thresholds to generate these variables, this process might be subject to interpretation and bias. Finally, although this first regional assessment provides further insights into challenges and strategies to strengthen community health systems, further research linking the reduction of bottlenecks and outcomes is warranted. In-country exercises may have taken place without counter-balancing independent views (civil society or nongovernmental organizations). Despite these constraints, the results are in line with previous health systems assessments conducted between 2010 and 2017^14^^,^^25^^,^^29^^,^^67^ using a quantitative bottleneck analysis tool to systematically assess bottlenecks on the basis of influential work by Tanahashi and Piot.^68^

### A Way Forward for All

The multicountry bottleneck analysis workshops provided an opportunity to engage country teams in identifying and prioritizing context-specific barriers to strengthen community health systems. Following this in-country exercise in 2019, countries such as Niger, Côte d'Ivoire, Sierra Leone, Mali, Burkina Faso, and Gambia have organized further dialogue with national experts and key stakeholders to review and revise their community health policy or strategy. The solutions proposed by the country teams could inform technical assistance needs and serve as a basis for further dialogue for countries to implement evidence-based, data-driven community health programs and build resilience. Table 5 highlights further actions needed to strengthen community health systems in the Community Health Roadmap countries. We must galvanize efforts to mobilize resources for effective PHC, which is fundamental to mortality reduction and reliant on strong community health systems to expand access to services.^69^

## CONCLUSION

In the context of PHC revitalization, addressing the regional situation of accelerating the reduction of maternal, neonatal, and child deaths by 2030 requires integrated, equity-focused, and multisectoral strategies, as well as strengthened community health systems.^1^ This article highlights bottlenecks and a way forward to optimize community health systems in one of the poorest regions in the world. In WCA, strengthening community health systems, as part of PHC revitalization efforts, should focus on increasing health financing and innovative investments, strengthening the logistics management system, and fostering community ownership and partnerships. Countries with high U5MR should also reinforce integrated service delivery through innovative approaches. Government actions galvanized by global and regional ongoing initiatives should be sustained to ensure that no one is left behind. Strong community health systems are fundamental to improve PHC services and move toward universal health coverage.

## Supplementary Material

20-00377-Simen-Kapeu-Supplement.pdf

## Translation

**En Français**

**Galvanisation de l'action en faveur des soins de santé primaires: analyse des goulots d'étranglement et stratégies pour renforcer les systèmes de santé communautaire en Afrique de l'Ouest et du Centre**

**Résultat Clé**

- Le financement de la santé, les produits et technologies médicaux essentiels, ainsi que l'appropriation et les partenariats communautaires sont apparus comme les goulots d'étranglement majeurs ou les plus critiques au renforcement des systèmes de santé communautaires, quel que soient les tendances de la mortalité.
- Les progrès impulsés par les pays sont possibles. Les stratégies potentielles pour lever les goulots d'étranglement comprennent l'augmentation de l'allocation budgétaire nationale et la mise à profit de mécanismes de financement innovants en faveur des soins de santé primaires (SSP), l'intégration des systèmes de chaîne d'approvisionnement et le renforcement de la mise en œuvre des politiques avec les communautés et les gouvernements locaux.
- Les pays ayant des taux de mortalité infantile élevés devraient améliorer la prestation des services grâce à une meilleure intégration.

**Implications Clé**

- Les pays doivent saisir les opportunités pour renforcer systématiquement les systèmes de santé communautaire dans leurs efforts pour atteindre la couverture sanitaire universelle.
- Nous devons galvaniser les efforts pour mobiliser les ressources pour des SSP efficaces, qui reposent sur des systèmes de santé communautaires solides pour élargir l'accès aux services et ne laisser personne sur le bord du chemin.
- La programmation intégrée à base communautaire devrait être renforcée pour en retour renforcer la résilience, la surveillance des maladies et les réponses rapides aux crises sanitaires, y compris celles dues aux maladies infectieuses comme la COVID-19.

**RÉSUMÉ**

**Introduction:** L'engagement renouvelé en faveur des soins de santé primaires (SSP) offre l'opportunité de renforcer les systèmes de santé en Afrique de l'Ouest et du Centre. Bien que des interventions peu onéreuses et efficaces, fondées sur des données probantes, pouvant prévenir jusqu'à un tiers des complications et des décès maternels, néonatals et infantiles avec une couverture universelle aient été identifiées, plus de 50% des personnes vivant dans les zones rurales ou issues de familles pauvres, dans des contextes aux ressources limitées, n'ont toujours pas accès à ces interventions simples. Cet article vise à évaluer les goulots d'étranglement et à proposer des solutions pour renforcer les systèmes de santé communautaire pour l'atteinte de meilleurs résultats.

**Méthodes:** Nous avons mené une analyse systématique multi-pays des goulots d'étranglement et proposé des solutions pour renforcer les systèmes de santé communautaire à travers une série d'ateliers collaboratifs dans 22 pays d'Afrique de l'Ouest et du Centre. Les pays ont été classés en fonction de leur taux de mortalité des moins de 5 ans pour évaluer les spécificités liées aux défis identifiés (> 75 décès pour 1 000 naissances vivantes, 25 à 75 décès pour 1 000 naissances vivantes et <25 décès pour 1 000 naissances vivantes). Nous avons également examiné les données existantes sur certaines interventions traceurs du système de santé pour analyser les profils des pays.

**Résultats:** Les goulots d'étranglement identifiés comme sévères ou très sévères étaient liés au financement de la santé (19 pays soit 86%), aux technologies et produits médicaux essentiels (16 pays soit 73%), à la prestation de services de santé intégrés (14 pays soit 64%) et à l'appropriation et aux partenariats communautaires (14 pays ou 64%). Seule la prestation de services intégrés a été déclarée comme un défi majeur par les pays avec un taux de mortalité des enfants de moins de 5 ans élevé. La question des ressources humaines pour la santé communautaire était l'un des défis les moins signalés. Après un examen approfondi des progrès effectués par les pays, nous avons proposé des stratégies pour renforcer les systèmes de santé communautaire.

**Conclusion:** En AOC, le renforcement des systèmes de santé communautaires dans le cadre des efforts de revitalisation des SSP devrait se concentrer sur : l'augmentation du financement de la santé et des investissements innovants, le renforcement du système d'information et de gestion logistique, et la promotion de l'appropriation et des partenariats communautaires. Les pays avec un taux de mortalité infanto-juvénile élevé devraient également renforcer la prestation de services intégrées par des approches innovantes. Les actions gouvernementales, galvanisées par les initiatives mondiales et régionales en cours, doivent être soutenues afin que personne ne soit laissé sur le bord du chemin.

**Peer Reviewed**
