# Supplementary material for: Galvanizing Action on Primary Health Care: Analyzing Bottlenecks and Strategies to Strengthen Community Health Systems in West and Central Africa
Source: Glob Health Sci Pract. 2021 Mar 15;9(Suppl 1):S47–64. doi: 10.9745/GHSP-D-20-00377 (PMC7971379; doi:10.9745/GHSP-D-20-00377)
Supplement: 20-00377-Simen-Kapeu-Supplement.pdf [file 20-00377-Simen-Kapeu-Supplement.pdf]

Supplement to: Simen-Kapeu A, Reserva ME, Ekpini RE. Galvanizing action on primary health care: a regional analysis of bottlenecks and strategies to strengthen community health systems in West and Central Africa. *Glob Health Sci Pract*. 2021;9(Suppl 1). https://doi.org/10.9745/GHSP-D-20-00377

ANALYTICAL TOOL TO ASSESS BOTTLENECKS AND CORRECTIVE SOLUTIONS

Supplement to: Simen-Kapeu A, Reserva ME, Ekpini RE. Galvanizing action on primary health care: a regional analysis of bottlenecks and strategies to strengthen community health systems in West and Central Africa. *Glob Health Sci Pract*. 2021;9(Suppl 1). https://doi.org/10.9745/GHSP-D-20-00377

A. DESCRIPTION OF THE TOOL

The tool aims to help countries conduct **a systematic analysis of the bottlenecks or barriers** that impede the effective implementation of primary health care, and to **identify remedial solutions**.

For all sections, the bottleneck analysis is mainly evaluated according to health system building blocks: leadership and governance, financial resources, health personnel, essential medical technologies and products, health service delivery (intervention, quality, integration, supervision), health information system, ownership and community participation. During the analysis, the degree / level of severity of the bottleneck must be defined.

B. GENERAL INSTRUCTIONS

Collection of information: Relevant documents and data sources to be collected include strategies / plans / policies / guidelines and national standards for reproductive, maternal, newborn and child health (RMNCH), national policies, guidelines and standards for reproductive health. IMCI, periodic reports, reviews, MNCH needs assessment and existing national survey data. For example, countries that have already done the global analysis of RMNCH interventions and commodities will have important information that can be used to provide background information and pre-fill the tool. Additional data will be available through the DHS / MICS, SARA, countdown, EmONC and other available MNCH surveys.

Analysis of information: A workshop or a regular technical meeting can be organized to carry out this analysis. Participants will identify health system barriers and solutions for scaling up child health and community health programs. The ranking of the severity of bottlenecks will be based on a subjective assessment that is subject to consensus in accordance with the prescribed scale.
Regarding the column on finding solutions in each section, each group will focus on the barriers that have been identified for each area of the health system. The group will identify possible solutions to overcome identified barriers. Solutions must be achievable (with clear steps) cost-effective, equity-focused and sustainable.

Validation of results: It is important that the analyzes, observations, and results recorded in the completed tool are reviewed and validated by the relevant Directions (Child Health Programs Directorate, Community Health Programs Directorate, Pharmacy Central and Logistics Branch). The list of experts (with signature) who have formed the working team must be attached when sending the completed tool. It would be useful to have a succinct national report summarizing the main obstacles to scaling up IMCI or institutionalizing community health as well as evidence-based solutions and actions to meet the challenges in a short term/medium period.

This information will serve as a programmatic basis for country support and tracking to accelerate the achievement of key outcomes for children.

Supplement to: Simen-Kapeu A, Reserva ME, Ekpini RE. Galvanizing action on primary health care: a regional analysis of bottlenecks and strategies to strengthen community health systems in West and Central Africa. *Glob Health Sci Pract*. 2021;9(Suppl 1). https://doi.org/10.9745/GHSP-D-20-00377

COMMUNITY HEALTH PROGRAMME

Section 1.1 Introduction to the context of the Community Health Programme

**elements**	**Detailed description** *(to be completed by countries)*
Current geographic coverage of the community health program	*Description (National ? regional ?) :*
Lists of partners (financing, implementation, etc.)	

Section 1.2 Bottleneck Analysis (and Underlying Causes) of the National Community Health Programme

**Areas**	**Essential elements to look for**	**Observed deficiencies and especially the underlying causes** *(which elements are missing ? Why ?)*	**S olutions proposed to solve the identified bottlenecks**
Legislation / policies	1. The validated and valid **Community Health Policy** takes into account :<br>a) Integrated management of childhood illnesses (malaria, pneumonia, diarrhea) ?<br>b) Standard Profile of the Community Health Worker (CHW) or equivalent defined ?<br>c) Clearly defined governance and community partnership framework ;<br>d) Authorization to care for sick children by CHW with antibiotics, ORS, Zinc, ACT ?<br>e) Integration of additional interventions is taken into consideration (integrated package)?<br>f) Policy that supports home visits by the CHW, including newborn care;		
	2. Is there a **strategic or operational** document that is budgeted and validated? Was it disseminated ?		
**Please make an overall assessment of your domain analysis** "Legislation / Policy" **- Select a single line :**<br>☐ **Documents (policy and strategies) available on child and community health**<br>☐ **Documents (policy and strategies) requiring few improvements** *(minimal revision required)*<br>☐ **Documents (policy and strategies) requiring major improvements** *(important amendments required)*			

Supplement to: Simen-Kapeu A, Reserva ME, Ekpini RE. Galvanizing action on primary health care: a regional analysis of bottlenecks and strategies to strengthen community health systems in West and Central Africa. *Glob Health Sci Pract*. 2021;9(Suppl 1). https://doi.org/10.9745/GHSP-D-20-00377

☐ **Policy and strategic documents not adapted to current context** *(full development required)*

**Areas**	**Essential elements to look for**	**Observed bottlenecks and especially the underlying causes** *(what elements are missing? Why?)*	**Proposed solutions to solve identified bottlenecks**
Governance / coordination at all levels	1. Is there a national coordinating committee for child health? Is there a national coordinating committee for community health? are they fully functional with defined roles and responsibilities?<br>2. Is the private sector engaged, on both coordination and implementation, of community health at all levels?<br>3. Is civil society engaged, on both coordination and implementation, of community health at all levels?		
	4. Is there a coordination system at the community level as " health management committee 'or equivalent (COGES, COSA, etc.)?<br>5. If the management committee exists at Community level, does it :<br>✓ The spots are clearly defined ?<br>✓ The members were elected by the community ?<br>✓ Reporting and performance evaluation mechanisms are in place and functional ?<br>✓ Collaboration with the health center is effective ?<br>✓ The committee is engaged in health planning activities at the local level and in the activities of CHWs (or equivalent)		

P lease make an overall assessment of your domain analysis " coordination **» - Select a single line :**

☐ **Coordination in place and functional at national and decentralized level**
☐ **Functional coordination at all levels but presents some challenges of implementation** *(2 out of 5 elements away s)*
☐ **Coordination is not functional and presents major challenges at the communal** *(4 out of 5 elements are absent)*
☐ **Coordination inadequate / not in place at all levels**

**Areas**	**Essential elements to look for**	**Observed bottlenecks and especially the underlying causes** *(what elements are missing ? Why ?)*	**Proposed solutions to solve identified bottlenecks**
Budgeting and funding community health	1. Is there a budget line for Community health?<br>2. Funds for community health are also generated by the government (funds disbursed)? What percentage ?<br>3. The Community Health **strategic** plan has is developed and budgeted?		

Supplement to: Simen-Kapeu A, Reserva ME, Ekpini RE. Galvanizing action on primary health care: a regional analysis of bottlenecks and strategies to strengthen community health systems in West and Central Africa. *Glob Health Sci Pract*. 2021;9(Suppl 1). https://doi.org/10.9745/GHSP-D-20-00377

	4. The Community Health **operational** plan is developed, budgeted and funded?<br>5. A resource mobilization plan for community health is developed?<br>6. Is there an investment framework for community health ?		
**P lease make an overall assessment of your domain analysis "** Financing" **- Select <u>a single</u> line**<br>☐ **Adequate funding mechanism adequate** (government budget, funding sources)<br>☐ **The financing domain requires some improvements** *(only 2 out of 6 items are missing)*<br>☐ **The financing domain** re**quires** major **improvements** *(at least 4 out of 6 items are missing)*<br>☐ **Financing mechanism is inadequate / undefined / no national plan**			
**Areas**	**Essential elements to look for in the national supply and management chain (C N AG)**	**Observed bottlenecks and especially the underlying causes** *(what elements are missing ? Why ?)*	**Proposed solutions to solve identified bottlenecks**
Availability of drugs	1. National quantification for essential medicines for community health is annually done by the government ?<br>2. Does the national logistic information management system take into account the Community level? or is there a parallel system for the moment ?<br>3. Essential medicines for child health (ORS, Zinc, antibiotics, ACT, etc.) can be found in the national list of essential medicines ?<br>4. The procedures for the distribution and storage of products at Community level are clearly defined in a manual available to all in the field?		
	5. Is the country experiencing **periodical stock shortages** / stockouts of child health essential commodities in health centers or at community level? Is there an alert system in place? What medicines are more concerned?<br>6. Community health workers have a **kit** that includes the timer, thermometer, and records / registers? do they have a place to store the drugs?		
	7. The revolving fund/ cost recovery mechanism is in place and functional?		
**Please make an overall assessment of your domain analysis " National Supply Chain and Management (SCM), especially the community level " - Select <u>a single</u> line :**<br>☐ **The national SCM system (mainly community level) is functional**<br>☐ **The national SCM system (mainly community level)) requires improvements** *(2/7 elements are absent)*<br>☐ **The national SCM system (mainly community level))** r**equires significant improvements** *(4/7 elements are absent)*<br>☐ **The national SCM system (mainly community level)) is non-functional / need to be effectively designed**			

Supplement to: Simen-Kapeu A, Reserva ME, Ekpini RE. Galvanizing action on primary health care: a regional analysis of bottlenecks and strategies to strengthen community health systems in West and Central Africa. *Glob Health Sci Pract*. 2021;9(Suppl 1). https://doi.org/10.9745/GHSP-D-20-00377

**Areas**	**Essential elements to look for**	**Observed bottlenecks and especially the underlying causes** *(what elements are missing ? Why ?)*	**Proposed solutions to solve identified bottlenecks**
Availability of qualified human resources	1. Is there a national document that describes the types, roles and responsibilities of community health workers and their supervisors?<br>2. Is there a document on job description? on the recruitment process ?<br>3. Is there a national document that describes the training required for CHWs or equivalents? Are the material / training tools are available at all levels (national, district health center) for periodic consultation?		
	4. Do all CHW or equivalent types have employment contracts currently valid?<br>5. Is there a mechanism in place for the regular assessment of CHW and their supervisors performance? is it functional ?<br>6. Is there a document that outlines the CHWs supervision mechanisms? is it functional (documented) at the operational level? are the funds available to facilitate the supervision processes?<br>7. Is there a document that specifies the mechanisms of retention and motivation?		
**P lease make an overall assessment of your domain analysis " Human ressources** (RH) » **- Select a single line :**<br>☐ **The HR domain has adequate / functional system**<br>☐ **The HR domain requires improvements** *(2/7 elements are absent)*<br>☐ **The HR domain requires significant improvements** *(4/7 elements are absent)*<br>☐ **The HR domain is not well defined (strategic level) and implemented (operational level)**			
**Areas**	**Essential elements to look for**	**Bottlenecks observed and especially the underlying causes** *(specify which elements are missing? explain the underlying causes of implementation delays ?)*	**Proposed solutions to solve identified bottlenecks**
Availability of community services and geographic accessibility, including integration and multisectoral	1) To assess accessibility to CHW services, analyze if the **number of CHWs** or equivalents types trained and operational in the field according to the policy is sufficient in relation to national needs (population living more than 5km from the HF)?		
	2) Is the **reference and counter-reference system** defined in the policy document? Is the system in place (forms, cards, transport) and functional? Is the reference system built with local communities that contribute?		

Supplement to: Simen-Kapeu A, Reserva ME, Ekpini RE. Galvanizing action on primary health care: a regional analysis of bottlenecks and strategies to strengthen community health systems in West and Central Africa. *Glob Health Sci Pract*. 2021;9(Suppl 1). https://doi.org/10.9745/GHSP-D-20-00377

	3) Management of cases of malaria, diarrhea , and pneumonia at Community level :<br><br>3.a. Analyze the integration of case management of the three major childhood illnesses **at Community level :**<br>✓ *This integration is allowed in the policy ?*<br>✓ *In the field, CHWs or equivalents take care of the three diseases? or some CHW or equivalents are designated for the detection and management of malaria cases only ?*<br>✓ *CHWs are equipped and provide a report on the number of cases followed at home for the 3 diseases?*		
	3.b For the implementation of Community IMCI , the country is in :<br>*a) planning phase and working on the development of national normative documents?*<br>*(b) pilot / project phase in selected areas?*<br>*c)* scaling-up *phase* ?		
	4) Analyze the integration of **MNCH and TB / HIV** interventions at the community level : *defined in the policy? field staff trained? CHWs or equivalent are equipped and engaged in monitoring TB / HIV + patients? tools available? Availability of ARVs at PMTCT sites? Surveillance, detection, and follow -up of TB / HIV cases at the community level is performed by CHWs ? If this integration is not currently effective, is this planned for this year 2019?*		
	5) Analyze the integration of **MNCH** interventions **and immunization** at the community level: *defined in the policy? promotion of vaccination is done by CHWs? Follow-up of the lost to follow-Ups at the community level? CHWs are equipped and tools available with good tracking system and documentation ?*		
	6) Analyze the integration of **iCCM** interventions and **nutrition** at Community level (prevention, detection of malnourished cases, growth monitoring , referral and home monitoring): *(Defined in the policy? field staff? CHW are equipped? tools available?)*		
	7) Analyze the integration of MNCH interventions **and birth registration** at Community level: *Defined in the policy? CHWs or equivalent know the mechanism to follow and practice it? Search for non-*		

Supplement to: Simen-Kapeu A, Reserva ME, Ekpini RE. Galvanizing action on primary health care: a regional analysis of bottlenecks and strategies to strengthen community health systems in West and Central Africa. *Glob Health Sci Pract*. 2021;9(Suppl 1). https://doi.org/10.9745/GHSP-D-20-00377

	registered children is effective and documented? tools available?		
	8) How is the expansion **of sexual and reproductive services for teenagers at the** primary care level**:**<br>*a) planning phase and development of national normative documents?*<br>*(b) pilot / project phase in areas?*<br>*c) scaling-up phase ?*		
	9) How is the expansion of **hygiene and sanitation interventions** at the community level **(**drinking water management, systematic handwashing, maintaining a clean environment):<br>*a) planning phase and development of national normative documents?*<br>*(b) pilot / project phase in selected areas?*<br>*c) scaling-up phase?*		
	**11)** How is the expansion of **Nurturing care/Early childhood Development** (plus community child care system) primary care level:<br>*a) planning phase and development of national normative documents?*<br>*b) pilot / project phase ?*<br>*c) scaling-up phase ?*		
	**12)** How is the expansion of **Social protection for the care of the poor and vulnerable at the primary care level** (health insurance system, cash transfer system, family / social support for the disabled, etc) :<br>*a) planning phase and development of national normative documents?*<br>*b) pilot / project phase ?*<br>*c) scaling-up phase?*		

P lease make an overall assessment of your analysis of the domain - select a single line:

- ☐ **Geographic accessibility and availability of services adequate**
- ☐ **Need some improvements** *(Only one third out of 11 elements are not implemented)*
- ☐ **Requires significant improvements** *(2/3 out of 11 elements are not implemented)*
- ☐ **Geographic accessibility / availability of integrated services are inadequate / no effective / nonfunctional**

**Areas**	**Essential elements to look for**	**Observed bottlenecks and especially the underlying causes** *(what elements are missing ? Why ?)*	**Proposed solutions to solve identified bottlenecks**
Health information system - community level (including monitoring and evaluation)	1. Is there a detailed operational document for monitoring and evaluation of the CHW programme?<br>2. Is the community health information system in place and functional?<br>3. Does the national health information management system take into account the		

<table>
<tr><td></td><td>Community level ? if not why ? is there a non-integrated system for the moment?
4. Is there information on the management of cases of illness in children and the number of children treated specifically for <u>malaria, diarrhea, and pneumonia</u> in the national system ?
5. Is patient follow-up documented? on cards or registers ?</td><td></td><td></td></tr>
<tr><td colspan="4">Please make an overall assessment of your HMIS domain analysis - select <u>a single line</u> :
o Community HMIS is functional, integrated into national system, and adequate (no obstacles)
o Community HMIS requires some improvements (2/5 elements not effective)
o Community HMIS requires significant improvements (3/5 elements are not functional)
o Community HMIS is non-functional (under development)</td></tr>
<tr><td>Areas</td><td>Essential elements to look for</td><td>Observed bottlenecks and especially the underlying causes (what elements are missing ? Why ?)</td><td>Proposed solutions to solve identified bottlenecks</td></tr>
<tr><td>Quality of services (supervision, equity, ethics, performance, respect of standards)</td><td>1. Link between the health center and the community: Does the health center have trained staff able to provide IMCI (3 diseases)? Does the health center effectively coordinate the implementation of CHW programme? Is the role of the health center clearly defined? Is there a monthly or regular interaction between the health center staff and the community around the CHW work?

2. The supervision of the health center is effective and documented: supervision guide available? performance assessment tools available? Effective and documented follow-up?

3. The algorithms and standards for the management of the 3 diseases (not only one) are available in health centers?

4. The services provided are equitably directed to the most vulnerable or hard-to-reach or poor populations (have they been identified with the community through a process? Is there a defined mechanism to support them)?</td><td></td><td></td></tr>
<tr><td colspan="4">Please make an overall assessment of your domain analysis " quality of care »:
☐ Implementation of a quality service effective and documented
☐ Need some improvements (some obstacles observed) – 2 out of 4 elements not covered)
☐ Need important improvements (major obstacles observed – 3 out of 4 elements not covered)
☐ Implementation of quality (including equity) processes inadequate/non effective</td></tr>
<tr><td>Areas</td><td>Essential elements to look for</td><td>Observed bottlenecks and especially the underlying causes (what</td><td>Proposed solutions to solve identified bottlenecks</td></tr>
</table>

Supplement to: Simen-Kapeu A, Reserva ME, Ekpini RE. Galvanizing action on primary health care: a regional analysis of bottlenecks and strategies to strengthen community health systems in West and Central Africa. *Glob Health Sci Pract*. 2021;9(Suppl 1). https://doi.org/10.9745/GHSP-D-20-00377

		elements are missing ? Why ?)	
Community involvement	1. Existence of **operationnel guide** on community engagement ?<br>2. Are there any **social accountability and citizen engagement** mechanisms in place and operating at the community level ?<br>3. The community plays an important role in supporting the CHW (ie discussing its role or goals, providing regular feedback) and helping to establish CHW as a community leader ?<br>4. Community leaders interact continuously within a <u>defined framework</u> with CHWs on health issues using data collected by CHWs ?<br>5. CHWs uses existing multisectoral community structures (eg, health committees, community meetings) for logistics, reference, or other ?<br>6. The group of youths, women's groups or other community groups are active extensions in support of CHW work?<br>7. The community interacts with the CHWs / equivalents and their supervisors during visits to provide feedback and resolve issues?<br>8. A wide range of community members play a role in planning the CHW program and provide feedback to the health center ?		
**After answering the previous questions, please make an overall assessment of your domain analysis " community engagement and partnerships » - please select <u>only one</u> <u>line</u> :**<br>☐ **Community involvement is effective and adequat**<br>☐ **Community involvement requires some improvements** *(3/8 elements are absent)*<br>☐ **Community engagement requires significant improvements** *(5/8 elements are absent)*<br>☐ **Community involvement is not effective**			

Supplement to: Simen-Kapeu A, Reserva ME, Ekpini RE. Galvanizing action on primary health care: a regional analysis of bottlenecks and strategies to strengthen community health systems in West and Central Africa. *Glob Health Sci Pract*. 2021;9(Suppl 1). https://doi.org/10.9745/GHSP-D-20-00377

Supplement TABLE 1. Country Self-reporting of Common Bottlenecks (Any Severity Level) to Scale Up the Community-Based Health Care in 22 Countries in West and Central Africa, 2019

Health system areas	Common Bottlenecks Identified by at Least 11 of 22 Country Teams During Workshops, Whatever the Grading of the Health System Areas	No. Countries
Leadership, governance - coordination	No national coordination committee/ national coordination committee in place but not functional	12
	No partnerships with the private sector (coordination, implementation of the CHW program)	20
	No involvement / no partnerships with the civil society (coordination, implementation)	11
	Community-based coordination structures in place but their functionality is a challenge	13
Health financing	No budget line for community health /PHC; National community health plan not budgeted, or plan budgeted but not validated	13
	Inefficiencies in financial flows within the health system (e.g., skewed allocation of funds to urban areas and specialized care, or from national to decentralized level); inadequate harmonization of external funds/aids from partners	13
	Lack of resource mobilization plan/ strategy for community health programs	20
	No investment plan for community health	12
Essential medical products and technology (tracer products are child lifesaving commodities: amoxicillin, ORS, zinc, ACT)	Policy and governance:<br>The national logistics management information system does not integrate the community level; the community-based management information system is parallel or developed for specific diseases; procedures not defined for the distribution and storage of products at community level (no manual)	 13
	Product quality and patient safety:<br>No national drug control laboratory certified by a standard accreditation agency<br>No drug quality monitoring system that meets the criteria (sampling, regular tests and actions in the past 12 months)<br>Patient safety monitoring system (pharmacovigilance) do not meet all defined criteria (questionnaire)	 11<br>13<br>17
	Procurement and availability of medicines<br>National quantification of drugs not systematically done every year (if done, it is mostly project-related); the quantification of community needs is not up to date or not taken into account during the national quantification<br>No monitoring and e-tracking system for the selected products from the first storage point to the health facility<br>National drug distribution system does not take into account the community level - parallel system linked to specific projects;<br>Supply chain management training not yet deployed in all public sector health facilities<br>Shortages of selected medicines (amoxicillin, ORS, zinc, ACT) have been registered in the past 12 months	 14<br><br>15<br>12<br><br>17<br>15
Human resources	No valid work contracts/agreements for CHWs	17
	Less than 70% of CHWs adequately trained to provide integrated community-based interventions as defined in national policy	17
	Nonfunctional retention and motivation mechanisms	12
	Insufficient number of CHWs or equivalent trained and operational according to the policy compared to national needs	15
	Referral and counter-referral system nonfunctional - and no contribution from local communities in most cases	11

Supplement to: Simen-Kapeu A, Reserva ME, Ekpini RE. Galvanizing action on primary health care: a regional analysis of bottlenecks and strategies to strengthen community health systems in West and Central Africa. *Glob Health Sci Pract*. 2021;9(Suppl 1). https://doi.org/10.9745/GHSP-D-20-00377

Health system areas	Common Bottlenecks Identified by at Least 11 of 22 Country Teams During Workshops, Whatever the Grading of the Health System Areas	No. Countries
Service delivery with integration, quality of care	No effective integration of MNCH and TB/HIV+ interventions (policy, implementation, tracking of patients, and resources)	13
	No effective integration of MNCH interventions and birth registration OR at pilot stage	11
	Very limited promotional and preventive adolescent sexual and reproductive health services at the community level	11
	Early childhood and education (including day care centers) interventions not in place or embryonic / pilot	19
	Supervision of CHWs by the health center is limited (less than 70% of CHWs covered) / not effective (no supervision guide / tools available / instability of staff) or poorly functional (no regular assessment – no feedback)	15
	Algorithms, protocols, and standards for integrated management of childhood illnesses not available at health centers/ no quality improvement mechanism	16
Heath information system	No community health monitoring and evaluation plan	11
	The community health information system is not fully functional; collection and analysis of community-based data is not regular	11
	The community health information system is parallel/or not yet integrated into the national health information system	11
	Algorithms, protocols, and standards for integrated management of childhood illnesses not available at health centers/ no quality improvement mechanism	16
Community ownership and partnership	Lack of community engagement plan/guide	13
	Mechanisms for social accountability and citizen engagement at the community level not in place or not functional	14
	Lack of framework to guide/maintain monthly community dialogue between CHWs and community leaders using monthly data collected for analysis and decision making	11
	Youth/women's groups or peer groups are not active extensions in support of CHWs	12
	Community does not interact with CHW (or equivalent) supervisor during visits to provide feedback and resolve issues	13
	Community members do not play an active/leadership role in CHW program planning	13

Abbreviations: ACT, artemisinin-based combination therapy; CHW, community health worker; ORS, oral rehydration salts; PHC, primary health care.

Supplement to: Simen-Kapeu A, Reserva ME, Ekpini RE. Galvanizing action on primary health care: a regional analysis of bottlenecks and strategies to strengthen community health systems in West and Central Africa. *Glob Health Sci Pract*. 2021;9(Suppl 1). https://doi.org/10.9745/GHSP-D-20-00377

Supplement TABLE 2. Highlights of Countries' Selected Recent Achievements to Strengthen Community Health Systems

Health system areas	Highlights of country progress or achievements	Examples of Countries[a]
Legislation, policies, governance, coordination	• Primary health care review/reform/plan; Primary Health Care Development Agency Act<br>• Development of: (1) national community health policies; (2) primary health care or community health plans<br>• Establishment of national community health or primary health care coordination committee	Nigeria, Gambia, Mali, Burkina Faso, Senegal, Liberia, Sierra Leone, Ghana
Health financing	• National investment cases (community health; RMNCH) and universal health coverage roadmap<br>• National health financing forum for resource mapping/ mobilization<br>• National Health Insurance financing model/ scheme / program<br>• Financial contribution of local collectivities / government through their annual investment plan.	Côte d'Ivoire, Guinea, Ghana, Gabon, Nigeria, Democratic Republic of Congo, Niger
Human resources	• Review / Definition of the CHW profile and package (included in the national community health policy)<br>• Harmonization of the remuneration of CHWs in targeted sites (Bilateral or multi-lateral grants; budget from municipalities);	Most Countries who recently reviewed their community health policy or plan
Service delivery with integration; quality of care	• Harmonization of CHW package of interventions in line with the basic package of essential health services;<br>• Implementation of child-friendly cities/ districts/community's models or community-led total sanitation communities to strengthen service integration<br>• Interoperability between health and civil registration systems, with the support of CHWs as civil registration agents in few countries.<br>• Adolescent health integrated into primary health care services	Mali, Equatorial Guinea, Guinea, Gabon, Liberia, Burkina Faso, Guinea-Bissau, Sao Tome and Principe, Benin, Senegal, Cap Vert, Gambia, Togo, Chad, Cameroun, Nigeria
Essential medical technology and products	• Review of community supply chain system – integration into national logistics management information system;<br>• Up-to date national guides for pharmaceutical supplies for the public health sector; strengthening of the quantification and regulatory framework: risk management and quality assurance procedures for supplies;<br>• Digital solutions to improve the supply chain system performance (e.g., e-tracker)<br>• Piloting of a modeling approach to improve the last mile (end users) distribution of commodities.	Côte d'Ivoire, Niger, Ghana
Health information system; monitoring and evaluation	• Integration of data from CHWs into DHIS2<br>• Piloting the use of digital solutions to enhance CHW program performance, including data reporting, client follow-up and health promotion messages	Liberia, Sierra Leone, Guinea, Togo, Benin, Democratic Republic of Congo, Guinea-Bissau
Community ownership and partnerships	• Community-led promotional and preventive interventions to increase awareness and behavioral change<br>• Engagement of community leaders and networks in decision making and monitoring enhanced through local government structures and local committees (mostly in selected areas)<br>• Engagement and leadership of the community health leaders, community groups, youths and associations in the CHW program; Community animation units to enhance community-based participatory approaches and promote essential family practices	Guinea, Senegal, Mauritania, Democratic Republic of Congo, Burkina Faso, Mali, Central Africa Republic

	• Community-led service delivery through community care sites (HIV screening, iCCM, hygiene, nutrition)<br>• Building partnerships with the local councils to enhance social accountability	

Abbreviations: CHW, community health worker; DHIS, district health information system; iCCM, integrated community case management of childhood diseases.

[a] This list of countries is not exhaustive; we have listed a few examples countries that have taken clear actions for a specific health system area.

Supplement to: Simen-Kapeu A, Reserva ME, Ekpini RE. Galvanizing action on primary health care: a regional analysis of bottlenecks and strategies to strengthen community health systems in West and Central Africa. *Glob Health Sci Pract*. 2021;9(Suppl 1). https://doi.org/10.9745/GHSP-D-20-00377

Supplement TABLE 3. Proposed Key Strategies, by Country Typology, to Enhance Current Efforts to Address Bottlenecks to Strengthen Community Health Systems in the Context of Primary Health Care

<table>
<tr><th colspan="2" rowspan="2">Health system areas</th><th colspan="2">Proposed Selected Strategies</th></tr>
<tr><th>Group 1 and 2: Countries with U5MR < to 75 deaths / 1,000 live births and with stronger health systems than Group 3 countries</th><th>Group 3: Countries with U5MR over 75 deaths/1,000 live births</th></tr>
<tr><td rowspan="4">Domains with Less Severe Bottlenecks (Country Self-grading)</td><td>Legislation, policies, governance, coordination</td><td colspan="2">• Review and sharpen national health/PHC/community health strategies and plans; Raise awareness on specific community health policy issues (parliament, champions) and strengthen linkages with national priorities
• Co-identify the most marginalized and vulnerable groups and co-develop priority health and social problems with communities and implement specific action plans; ensure the gender-responsiveness of all policies and plans
• Leverage / strengthen innovative partnerships (e.g., private sector, CSOs)
• Strengthen cross-departmental alignment of strategic priorities and the functionality of national and local coordination structures</td></tr>
<tr><td>Human resources</td><td>• Increase efficiency and maintain skills and competencies of integrated PHC teams (including CHWs), especially in remote and rural areas
• Work with local governments and other line ministries (social welfare, youth, education) to ensure long-term availability of CHWs (remote and rural areas)
• Offer career ladder /advanced education and work opportunities to practicing CHWs based on nationally defined criteria</td><td>• Work toward integrating CHWs as part of the PHC multi-disciplinary team and expand quality improvement mechanisms to increase CHW performance
• Implement national human resources strategies to increase availability and retain skilled providers in PHC facilities
• Strengthen and support community providers through regular supportive supervisory visits by health facility staffs
• Define the CHW financial commensurate with the job requirements; provide written contact agreements to CHWs</td></tr>
<tr><td>Service delivery with integration and quality of care</td><td>• Use innovative approaches to reach universal coverage of lifesaving interventions (targeting poor and marginalized communities)
• Develop quality improvement models to improve services provided at the PHC facilities (clinical, outreach and mainly community)</td><td>• Develop an integrated general package of interventions for CHWs, in line with the national basic package of services, taking into consideration CHW workload (as per national standards)
• Reinforce supervision and mentoring for CHWs to improve quality of services delivered to communities and linkages with health facilities
• Implement innovative approaches to serve the most marginalized groups and reduce gender inequalities and health inequities
• Strengthen referral and counter-referral system in close collaboration with communities</td></tr>
<tr><td>Health information system / monitoring and evaluation</td><td>• Scale up the use of innovative technologies for better health management information system
• Policy and system research should evaluate strategies on scalability, sustainability and cost-effectiveness to guide decision making</td><td>• Ensure community health information system is integrated in the national/district health information system
• Develop metrics for effective CHW program implementation that could guide financing support; ensure an adequate monitoring and evaluation system is in place to assess/ review progress for better results
• Develop and implement innovative and digital approaches to strengthen data collection and use at the community level</td></tr>
</table>

Supplement to: Simen-Kapeu A, Reserva ME, Ekpini RE. Galvanizing action on primary health care: a regional analysis of bottlenecks and strategies to strengthen community health systems in West and Central Africa. *Glob Health Sci Pract*. 2021;9(Suppl 1). https://doi.org/10.9745/GHSP-D-20-00377

Domains self-graded by most country teams as severe/very severe	Health financing	• Plan transition from internationally financed to domestically financed programs: by developing long-term CHW plans (1) Increase domestic allocation for primary health care, including community health for program expansion; (2) leverage innovative funding mechanisms: education and economic growth programs; co-financing/ matching funds, trust funds with private sector, human capital bonds<br>• Scale-up alternative provider payment systems for PHC, including performance-based financing<br>• Generate evidence on best financing models for the national CHW program	• Develop an investment case and return of investment analyses for community health programs;<br>• National health financing strategies should consider CHWs systems within a broader framework of financing for Universal Health Care;<br>• Develop clear gap analyses and financing pathways for costed CHW programs by leveraging domestic/ international resources as well as new funding sources including matching grants, co-investment with local collectivities or governments, disease surveillance preparedness and global security<br>• Reduce community-based program fragmentation through joint budgeting to maximize gains<br>• Prioritize the use and benefits of public funds committed to health across socioeconomic groups to make all levels of health financing systems more accountable and transparent; implement and expand pro-poor legislation and strategies (e.g., vouchers, community-based health insurance schemes)
	Essential medical technology and products	• Deploy digital systems to support different supply chain functions, including forecasting software/systems to manage seasonal requirements<br>• Expand supply chain management trainings in all health facilities including CHWs, in close collaboration with the private sector)<br>• Strengthen quality assurance mechanisms (e.g., certified national drug control laboratory)	• Integrate community health supplies into the national logistics management information system<br>• Allocate funding for procurement of lifesaving commodities to be used to deliver community-based interventions<br>• Create policies to facilitate investments in local production for essential lifesaving medicines<br>• Improve forecasting/quantification systems and tools for existing products developed
	Community ownership and partnerships	• Planning: (1) develop national community engagement plan/guide to better orientate responses to local needs; (2) develop community linkages and conduct community-led advocacy and research to better informed existing policies<br>• Governance and coordination: (1) enhance community representation and voice in relevant governance and oversight mechanisms; (2) engage local authorities/councils and partner with community leaders, groups, CSOs, NGOs, youths during the planning, implementation, and monitoring of the CHW program<br>• Implementation and monitoring: (1) establish community-based monitoring systems to enhance information sharing; (2) establish functional mechanisms for social accountability (community scorecards, observatories) and citizen engagement with communities to solicit feedback and strengthen quality programming; (3) develop partnerships with local organizations and networks and build their capacities to reinforce implementation of social and behavioral change, social marketing, community mobilization, promotional and preventive integrated community-based interventions through innovations (e.g., mHealth)	

Abbreviations: CHW, community health worker; CSO, civil society organizations; NGO, nongovernmental organizations, PHC, primary health care; U5MR, under-5 mortality rate.
